# Long term oncological outcome of thymoma and thymic carcinoma – an analysis of 235 cases from a single institution

**DOI:** 10.1371/journal.pone.0179527

**Published:** 2017-06-20

**Authors:** Yen-Chiang Tseng, Yen-Han Tseng, Hua-Lin Kao, Chih-Cheng Hsieh, Teh-Ying Chou, Yih-Gang Goan, Wen-Hu Hsu, Han-Shui Hsu

**Affiliations:** 1 Division of Thoracic Surgery, Department of Surgery, Kaohsiung Veterans General Hospital, Kaohsiung, Taiwan; 2 Institute of Clinical Medicine, National Yang-Ming University, National Yang-Ming University School of Medicine, Taipei, Taiwan; 3 Division of Thoracic Surgery, Department of Surgery, Taipei Veterans General Hospital, Taipei, Taiwan; 4 Department of Chest Medicine, Taipei Veterans General Hospital, Taipei, Taiwan; 5 Department of Pathology and Laboratory Medicine, Taipei Veterans General Hospital, Taipei, Taiwan; 6 Institute of Emergency and Critical Care Medicine, National Yang-Ming University, Taipei, Taiwan; Baylor College of Medicine, UNITED STATES

## Abstract

**Background and objectives:**

Thymoma has a variable long-term oncological outcome after surgical resection. Survival and tumor recurrence were analyzed to determine the predisposing factors for tumor recurrence.

**Methods:**

A total of 235 patients who underwent surgery for thymoma or thymic carcinoma from December 1997 to March 2013 were analyzed using Masaoka staging system and World Health Organization (WHO) histological classification. Surgical intervention included extended thymothymectomy via median sternotomy and thymomectomy via thoracotomy/ video-assisted thoracoscopic surgery (VATS).

**Results:**

The median duration of follow-up was 105 months (12–198 months). Among these 235 patients, recurrence was observed in 25 patients (10.7%). according to Masaoka stage I, IIA, IIB, III, IVA, IVB, recurrence rates were 1/65(1.5%), 8/106(7.5%), 1/32(3.1%), 6/20(30.0%), 8/10(80.0%), 1/1(100.0%), respectively. Disease or treatment-related mortality was observed in 13 patients. Overall survival rate was 94.4%. After univariate analysis, predisposing factors for tumor recurrence included Masaoka stage, WHO histologic type, tumor size, adjuvant therapy and margin status.

**Conclusions:**

Due to the indolent behavior of thymoma, tumor recurrence appears to be a better assessment of oncological outcome rather than survival. Factors associated with tumor recurrence include Masaoka stage, WHO histologic type, tumor size, adjuvant therapy and margin status.

## Introduction

Thymoma is the most common mediastinal tumor, accounting for approximately 20% of all mediastinal masses and up to 50% of all anterior mediastinal masses [1]. The incidence of thymoma is estimated at 1.5 per million persons in the United States and 6.3 per million in Taiwan [2,3]. The Masaoka staging system, as modified by Koga et al. in 1994, is the most popular staging system [4,5]. Previous studies have shown that this staging system is a good predictor of tumor recurrence [6]. According to this system, staging is based on level of invasion and is divided into stage I, IIA, IIB, III, IVA, and IVB. Another prognostic factor for thymoma, the World Health Organization (WHO) classification, has further subdivided thymoma into six different types (A, AB, B1, B2, B3, and thymic carcinoma) according to tumor histology [7].

Despite the use of these two staging methods, the predisposing factors for thymoma recurrence are still unclear. Long-term oncological outcomes after surgical resection also vary across studies. We, therefore, reviewed the experiences of a single institution in the treatment of thymomas and thymic carcinoma over a 16-year period to determine the predisposing factors influencing tumor recurrence. In addition, the prognostic factors affecting long-term survival, as determined by the WHO classification and Masaoka staging system, were also examined.

## Materials and methods

### Patient characteristics

The Institutional Review Board at the Taipei Veterans General Hospital approved this study and granted an exemption from informed consent (201208010BC).

A total of 246 patients underwent surgery for thymoma or thymic carcinoma at Taipei Veterans General Hospital from December 1997 to March 2013. The treatment principles followed by our institution are according to the NCCN guideline and the decision of multi-discipline team. Patients who received neoadjuvant chemoradiation (n = 4), who had undetermined WHO histological type owing to unavailability of slide specimens (n = 4), and patients who had only open biopsy or port-A insertion (n = 3) were excluded. Finally, 235 patients were selected for analysis.

Surgical intervention included extended thymothymectomy via median sternotomy and thymomectomy via thoracotomy or video-assisted thoracoscopic surgery (VATS). VATS was performed via a thoracoscopically-guided anterior minithoracotomy through a 2–3 cm working port.

For those patients undergoing thymothymectomy, surgery was performed as previously described [8] via a median sternotomy. Briefly, following entry into the mediastinum, the pleura was opened on both sides, and the thymus, tumor and adjacent pericardial fat, was resected. The cervical extension of each lobe with the body of the gland was removed by gentle traction. The phrenic nerves were preserved throughout the procedure.

For patients treated with thoracotomy or VATS without thymectomy, they were placed in right or left lateral positions as previously described [8]. The tumor and some thymic tissue or perithymic fat was resected with a safe margin. The phrenic nerve was preserved during the procedure. In cases for which tumor seeding was identified, the patients also underwent pleural partial resection or electrocauterization.

Staging was performed using the new TNM staging system [9] and according to Masaoka stage [4], as modified by Koga et al. [5]. The histological classification was performed using the WHO histological typing of thymoma [7]. All slides of thymoma or thymic carcinoma were reviewed by two experienced pathologists (H-L K and T-Y C). When a tumor exhibited mixed histologic types, the tumor was classified according to the most histologically aggressive type present. For example, when the tumor had both B2 and B3 components, the tumor was classified as type B3. The R classification, which was adopted by the UICC, was used to document the presence or absence of residual tumor after treatment. Residual tumor may be localized in the area of the primary tumor and/or as distant metastases. R0 corresponds to resection for cure or complete remission, R1 corresponds to microscopic residual tumor and R2 reflects macroscopic residual tumor. In this study, we defined all IVA and IVB patients as R2 resection as a result of tumor seeding of pleural or pericardium.

Computed tomography (CT) of the chest was performed at 6-month intervals for the first 2 postoperative years and at 1-year intervals for the subsequent 3 years. After 5 years of follow-up without tumor recurrence, lifelong follow-up was recommended every 1 to 2 years.

### Statistical analysis

IBM SPSS statistical software version 22 for Windows (IBM, Armond, NY, USA) was used for data analysis. Continuous data were expressed as median with range. Continuous variables were analyzed by the independent *t*-test or the Mann-Whitney U test. To compare the frequencies between the two groups, Chi-square tests were applied for the univariate analysis. Overall survival and freedom from recurrence curves were estimated by the Kaplan–Meier method and compared by the log-rank test. A *p* value of less than 0.05 was considered statistically significant. The predisposing factors were analyzed using Cox regression tests for univariate analysis.

## Results

### Patient characteristics

The demographics and tumor characteristics of the 235 patients enrolled in this analysis are shown in Table 1. The median follow-up duration was 105 months (range: 12–198 months). The median age of all patients was 51 years (range: 20 to 85 years). More females [132 patients (56.17%)] than males [103 (43.83%)] were included in this study.

**Table 1:** Characteristics of 235 patients undergoing thymoma resection.

Variables	Number	Percentage (%)
Age, median (range), years	51.00 (20–85)	
Gender		
Male	103	43.83
Female	132	56.17
Surgical approach		
Median sternotomy	145	61.70
Thoracotomy	40	17.02
VATS	50	21.28
Extent of resection		
Thymomectomy	90	38.30
Extended thymothymectomy	145	61.70
Completeness of resection		
R0	228	97.02
R1	2	0.85
R2	5	2.13
Myasthenia Gravis (MG)		
With MG	75	31.91
Without MG	160	68.09
WHO histological types		
A	19	8.10
AB	69	29.36
B1	56	23.83
B2	50	21.28
B3	41	17.45
Masaoka stage		
I	65	27.66
II	138	58.72
III	21	8.94
IV	11	4.68
Tumor size, median (range), cm	6.0 (1.5–16.0)	
Adjuvant therapy		
None	133	56.60
Radiotherapy	99	42.13
Chemoradiation	3	1.28
Mortality		
Yes	13	5.56
No	221	94.44
Recurrence		
Yes	25	10.68
No	209	89.32

VATS: Video-assisted thoracoscopic surgery

MG: Myasthenia Gravis

The surgical approaches included median sternotomy, thoracotomy, and VATS. The definition of thymoma recurrence used in this study was proposed by ITMIG [10]. Most of the patients (228 patients, 93.44%) received R0 resection of thymoma or thymic carcinoma, and few patients received R1 or R2 resection as a result of more advanced stages. All 75 patients (31.91%) with myasthenia gravis received extended thymothymectomy. The median tumor size was 6.0 cm (range: 1.5–16.0 cm). Some patients received adjuvant radiotherapy or chemoradiation therapy after surgery.

### Recurrence and overall survival

**Fig 1:**
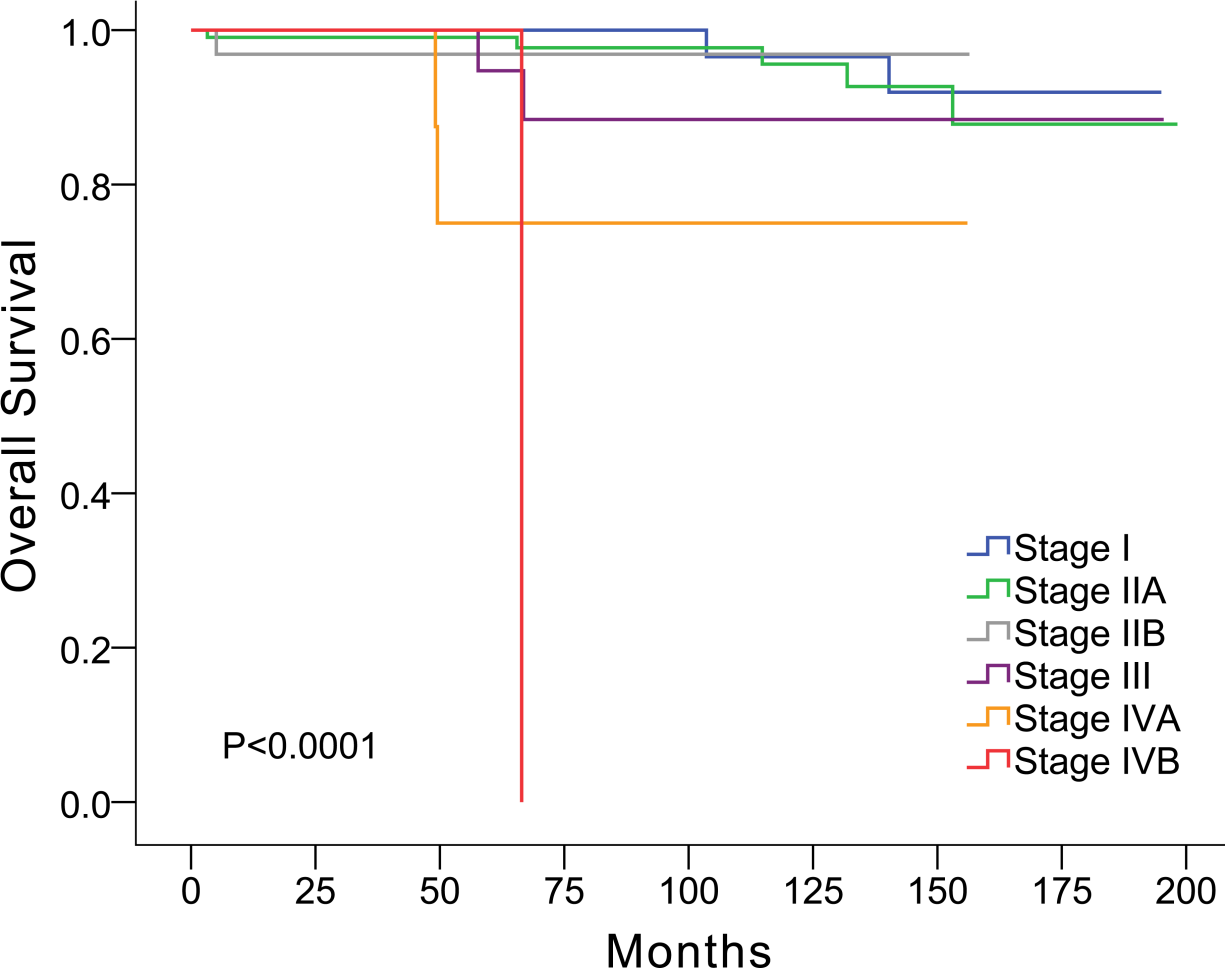
Overall survival correlated with Masaoka stage.

Of the 235 patients, recurrence was observed in 25 patients (10.7%; Table 1). The distribution of Masaoka stage in the thymoma subtypes is shown in Table 2. The recurrence rate over the median follow-up period of 105 months, according to Masaoka stage I, IIA, IIB, III, IVA, and IVB was 1/65 (1.5%), 8/106 (7.5%), 1/32 (3.1%), 6/20 (30.0%), 8/10 (80.0%), and 1/1 (100.0%), respectively. Local recurrence in the anterior mediastinum or adjacent pleura was noted in six patients. There were 12 patients with regional recurrence, which included pleural and pericardial nodules. There were 10 patients with distant recurrence including three bony metastasis, one with liver metastasis and six patients with intraparenchymal pulmonary nodules. Disease-related or treatment-related mortality was observed in 13 patients. The overall survival rate was 94.4% (Fig 1; Table 1).

**Table 2:** Distribution of Masaoka stage in the thymoma subtypes.

	WHO histological type						
Masaoka stage		A	AB	B1	B2	B3	Sum
	I	0/11	0/29	1/13	0/10	0/2	1/65 (1.5%)
	IIA	1/5	1/21	1/33	1/24	4/23	8/106 (7.5%)
	IIB	0/3	0/12	0/5	1/10	0/2	1/32 (3.1%)
	III	0	1/7	0/1	2/5	3/8	6/20 (30.0%)
	IVA	0	0	3/3	1/1	4/6	8/10 (80.0%)
	IVB	0	0	1/1	0	0	1/1 (100.0%)
	Sum	19	69	56	50	41	25/234 (10.7%)

### Predisposing factors of recurrence

Possible predisposing factors included for analysis were gender, age, Masaoka stage, WHO histology type, extent of resection (extended thymothymectomy or thymomectomy), median tumor size, adjuvant therapy, myasthenia gravis, postoperative myasthenia gravis, and resection margin status.

**Fig 2:**
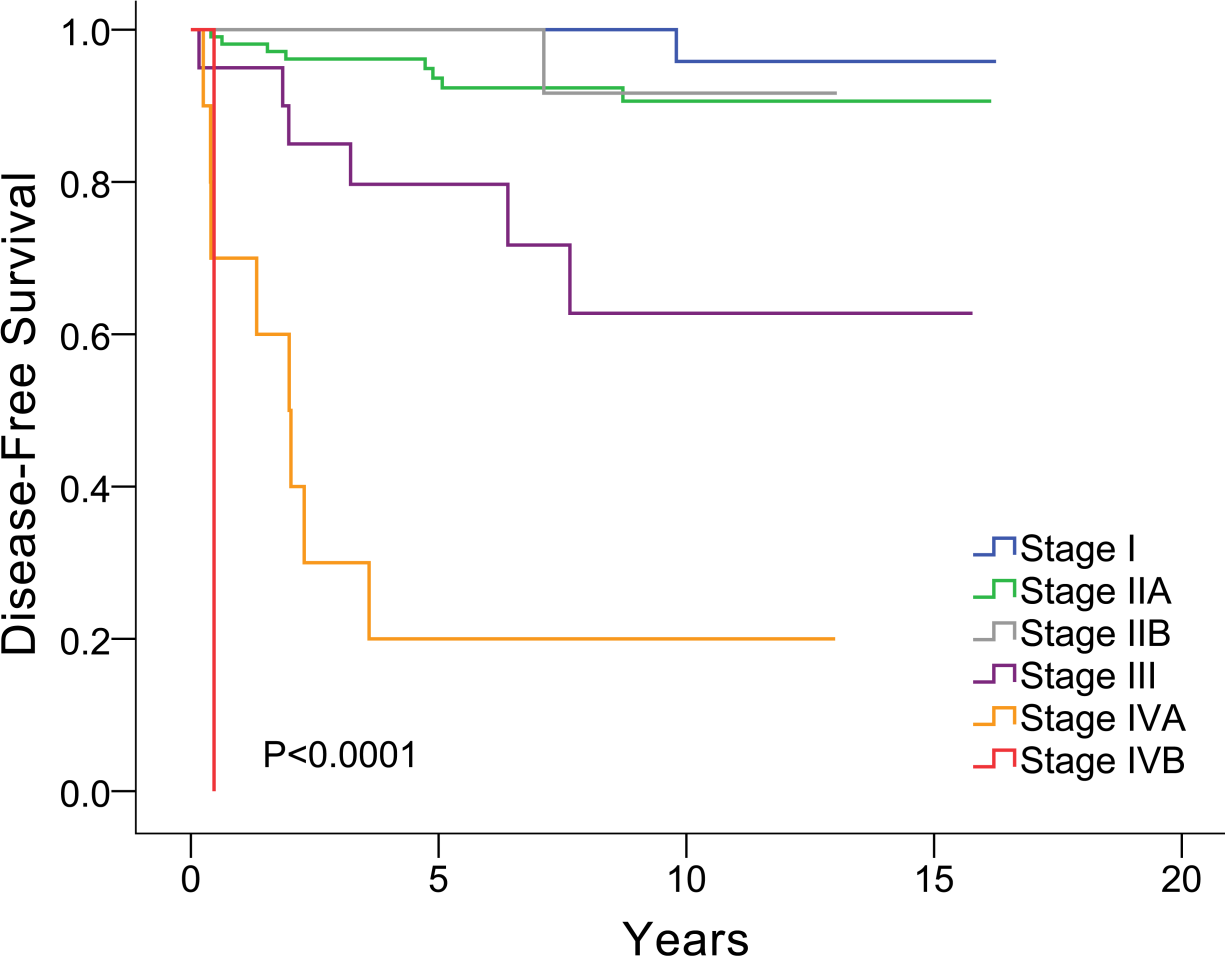
Correlation between Masaoka stage and disease free survival.

**Fig 3:**
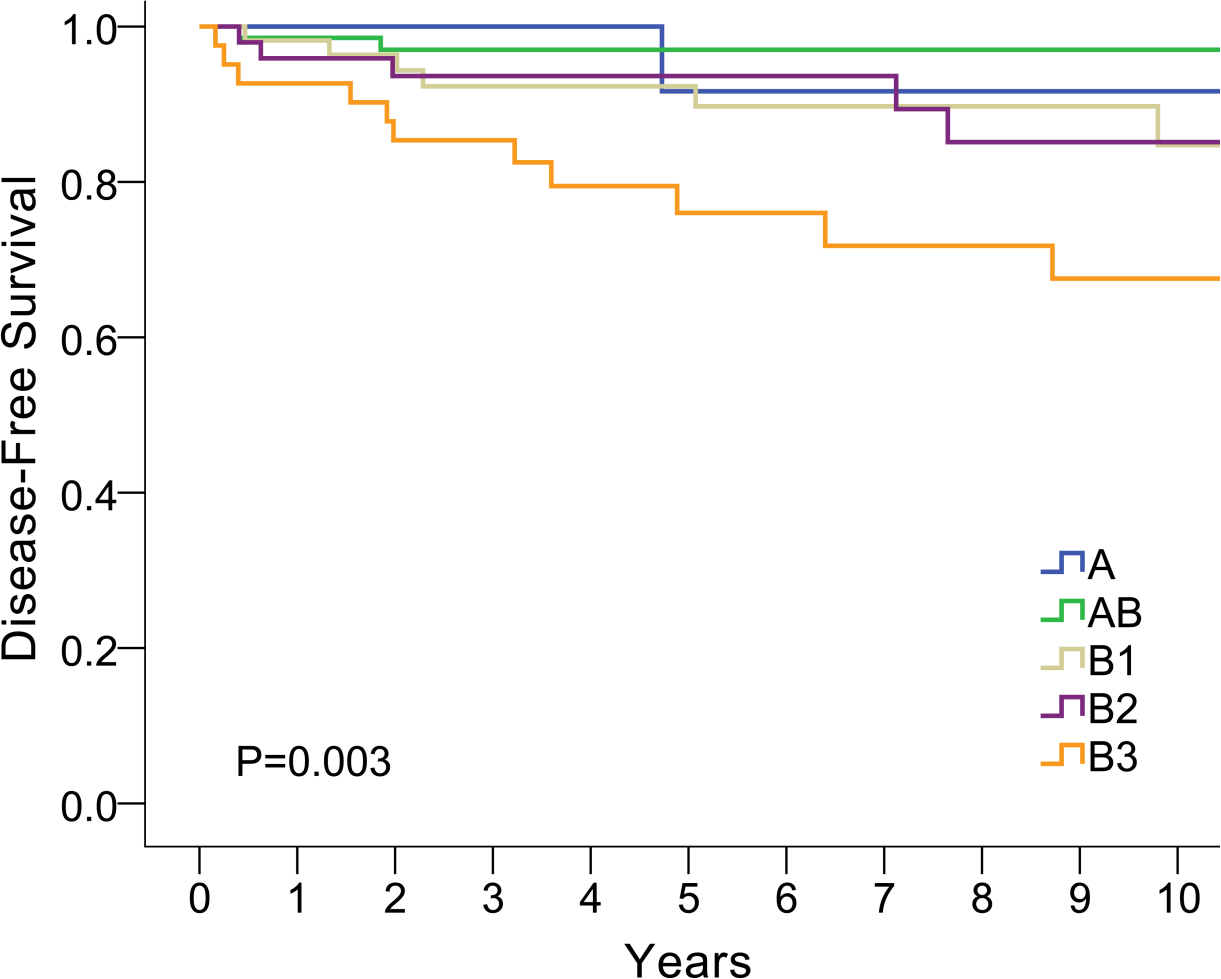
Correlation between WHO histology type and disease free survival.

From univariate analysis, six factors were associated with tumor recurrence, including Masaoka stage, TNM stage (8^th^ edition), combined WHO histologic type, with or without adjuvant therapy, R0 or R+ resection, and tumor size (Table 3). The correlations between Masaoka stage and disease-free survival and between WHO histological type and disease-free survival are plotted in Figs 2 and 3, respectively.

**Table 3:** Univariate Cox regression analysis to identify factors associated with recurrence.

	Univariate	
	HR (95% CI)	*P*-value
Gender		
F	ref	
M	0.71(0.31,1.6)	0.405
Age	1.00 (0.98, 1.03)	0.774
Masaoka Stage		
I	ref	
II (IIA and IIB)	4.23(0.54,33.36)	0.172
III	20.95(2.52,174.12)	0.005
IV (IVA and IVB)	113.65(14.24,907.17)	<0.001
New TNM stage		
I	ref	
II	4.59(1.23,17.12)	0.023
IIIa	4.31(1.33,14.01)	0.015
IIIb	19.5(4.2,90.47)	<0.001
IVa	41.35(15.8,108.17)	<0.001
IVb	114.2(12.77,1021.17)	<0.001
WHO histology type		
A	ref	
AB	0.49(0.04, 5.45)	0.565
B1	1.94(0.23,16.13)	0.540
B2	1.92(0.22,16.41)	0.553
B3	5.15(0.66,39.93)	0.117
Combined WHO histology types		
A/AB/B1	ref	
B2/B3	3.05(1.35,6.9)	0.007
Extent of resection		
Extended thymothymectomy	ref	
Thymomectomy	1.15(0.50,2.62)	0.745
Median tumor size	1.23(1.1,1.37)	<0.001
Adjuvant therapy (ref = No)	5.87(2.2,15.65)	<0.001
Myasthenia gravis (ref = No)	0.69(0.29,1.65)	0.402
Postoperative MG (ref = No)	1.4(0.63,3.12)	0.413
Margin status (ref = Free)	13.79(5.69,33.38)	<0.001

MG: Myasthenia gravis

## Discussion

Thymoma is an indolent tumor with no definitive staging system currently in use. To evaluate tumor behavior and the predisposing factors for tumor recurrence, we retrospectively analyzed all patients with thymoma who underwent surgical treatment at our institution over a 16-year period. According to Cox univariate analysis, we found that Masaoka stage, TNM stage, WHO histology type, size of tumor, and relative thoroughness of tumor resection were associated with tumor recurrence. These findings are consistent with previous studies concerning prognosis after thymoma resection [6,11–14].

Surgical resection is considered to be the mainstay of treatment for thymoma. Recent guidelines have recommended complete en‐bloc resection of the tumor with the entire thymus gland. At our institution, en bloc resection is recommended for advanced stage and for patients with myasthenia gravis. In non myasthenic patients with early thymoma, thymomectomy is considered an acceptable treatment, as discussed previously [8,15].

For patients with thymomas, incomplete resection (including R1 or R2 resection) was the predisposing factor for thymoma recurrence [6,13]. In our study, we also found that patients with R1 or R2 resection of thymoma had worse prognosis and higher recurrence rates than patients with R0 resection.

The role of adjuvant radiotherapy or adjuvant chemoradiation after surgery is debatable, particularly for stage II disease. Although increased overall survival has been reported with the use of adjuvant radiotherapy, most studies have combined stage II and stage III disease. Some studies have suggested that there may be little benefit of adjuvant radiotherapy in stage II disease given the low recurrence rate in this group of patients [16,17]. In our study, patients with adjuvant radiotherapy or chemoradiation had worse prognosis. This may be due to the fact that all stage IV patients and most stage III patients received adjuvant therapy. In the subgroup analysis, there was no survival benefit in stage II patients who underwent adjuvant therapy (p = 0.590) or for stage III patients who underwent adjuvant therapy (p = 0.858).

Although the Masaoka stage has long been shown to be the most important prognostic factor for thymic malignancy, several studies have found other factors as being important. For example, Fukui et al. [18] showed that tumor size is an important indicator. In addition, multivariate analyses by Fu et al. [19] identified R0 resection, Masaoka-Koga stage, and postoperative radiotherapy as significant prognostic factors of survival in thymic carcinoma patients. In the present study, univariate analysis identified that Masaoka stage, combined WHO histologic type, with or without adjuvant therapy, R0 or R+ resection, and tumor size were all associated with tumor recurrence, which is consistent with previous studies [20–22].

WHO histological type is another predisposing factor for tumor recurrence. Guerrera et al. [23] found that WHO histology type is an important prognostic factor of outcomes following thymomectomy. The histologic types B2 and B3 had higher recurrence rates than type A, AB or B1 in our study. Similar to our results, Ströbel et al. [6] reported that types A, AB, and B1 behaved in a benign fashion, while types B2 and B3 behaved in a more malignant fashion. Okumura et al. [24] also suggested that type B2 and B3 tumors were more malignant, in terms of tumor recurrence, compared with types A, AB, and B1. In our study, the histological type AB was the most frequently encountered type (28.27%), followed by B1 (22.95%), and B2 (20.49%). Margaritora et al. [25] analyzed 317 patient with thymomas (including some cases with WHO thymic carcinoma), and found that type B2 tumors were the most frequently observed (57.5%), followed by types B1 (19.2%) and AB (9.5%).

Based on the findings of the present study, we recommend doing contrast chest computed tomography (CT) and blood tests (e.g., CBC and serum biochemistry analyses) for postoperative surveillance in patients with thymic epithelial tumors. For patients with myasthenia gravis symptoms, Ach receptor antibody testing may also be required. Postoperative surveillance of thymic carcinoma may also include imaging and analysis of tumor marker levels, such as CEA and SCC.

Our study had several limitations, including its retrospective nature, its limited (105-month) median follow-up time, and the small number of thymic carcinoma patients. Also, multivariable analysis was not conducted due to the limited number of events. Since thymoma is an indolent tumor, a longer follow-up time may be needed. In addition, surgical procedures were performed according to tumor location and surgeon’s preferences, and some selection bias may have existed. Finally, this study included only resected thymoma or thymic carcinoma patients from a single institution. The size of our cohort may not have been sufficient to reach an appropriate conclusion. A case-matched or prospective, controlled study with a larger patient cohort is needed to confirm our findings.

In conclusion, as a result of the indolent behavior of thymoma, tumor recurrence appears to be a better assessment of oncological outcome compared with survival. The predisposing factors affecting tumor recurrence included stage, histologic type, thoroughness of resection, and tumor size. Longer follow-up time with a larger patient cohort is required to investigate the oncological behavior of thymoma or thymic carcinoma.
