# Correction: Long term oncological outcome of thymoma and thymic carcinoma - an analysis of 235 cases from a single institution

**DOI:** 10.1371/journal.pone.0185399

**Published:** 2017-09-20

**Authors:** Yen-Chiang Tseng, Yen-Han Tseng, Hua-Lin Kao, Chih-Cheng Hsieh, Teh-Ying Chou, Yih-Gang Goan, Wen-Hu Hsu, Han-Shui Hsu

The affiliation for the second author is incorrect. Yen-Han Tseng is affiliated with both **4** Department of Chest Medicine, Taipei Veterans General Hospital, Taipei, Taiwan and **7** National Yang-Ming University School of Medicine, Taipei, Taiwan.
